# Pleurisy

**Published:** 1893-01

**Authors:** E. S. McKee

**Affiliations:** Cincinnati, Ohio


### PLEURISY.

By E. S. McKEE,
Cincinnati, Ohio.

Fernet reports a case which he terms "typhoid pleurisy," in which was found in abundance the typhoid bacillus of Eberth. The patient manifested in addition to pleuritic symptoms the general signs of enteric fever except the rose spots. Mr Fernet was of the opinion that the pleurisy was the antecedent manifestation of the typhoid fever and that, like the latter, it was set up by the bacillus of Eberth.

Eberth's bacillus has been found in a hemorrhagic pleural effusion by Charvin and Royer. There cultures, very virulent in mice, gave rise in the guinea pig to bloody effusions in the pleura and peritoneum. Fernet has also found this bacillus in a case of sero-fibrinous pleurisy.

The etiology of primary pleurisy is described by Patella, as follows : There are fibrinous pleurisies due to the encapsulated micrococcus of Fræenkel ; pleurisies with abundant effusion, of exclusively tuberculous origin. In a tuberculous individual a pleurisy may sometimes take on a simple non-tuberculous form. There are also pleurisies of chemical origin, which are still insufficiently known. He thinks it belongs to chemical rather than bacteriological researches to determine the causes and nature of primitive pleurisies. In all cases when bacteriological research shows the presence of the diplococcus of Fræenkel we may hope for the spontaneous resorption of the exudation.

Renze has become convinced by his bacteriological researches that pleurisies were caused, according to the prevalent epidemic constitution, sometimes by the diplococcus of Frænkel and sometimes by the streptococcus; thus during the last epidemic of influenza, streptococcus pleurisies predominated. He admits the curability of tuberculous pleurisies. Bozzolo observes that the microbe of typhoid fever provokes pleurisy oftener than one would suppose.

The etiology of pleuritis, according to Patella, is due in certain acute cases to nothing else than infection through the diplococcus capsulatus lanceloatus of Frænkel. Some other pleurisies are tubercular alone.

Calcareous degeneration of the pleurisies reported by Poulaillon is a subject of great interest to the surgeon and physician, especially in relation to paracentesis and resection of the rib. Pathological research shows this is first developed through hyperplasia of the sub-endothelial connective tissues of the parietal layer of the pleura ; they are seldom, if ever, of purely pulmonary origin. Traumatism is the most prevalent cause of their development. Diagnosis by physical signs is seldom possible.

Pulmonary gangrene is given great prominence as a cause of putrid pleural effusions by James.

That encysted pleural effusions are quite frequent is maintained by Simon. He also claims that this encysting may take place either in the upper or lower parts of the chest.

Diaphragmatic pleurisy is reported by Rastamenla. The patient was at first supposed to be suffering from broncho-pneumonia. He had great dyspnœa, respirations 42, pulse 130, temperature 104°. A physical examination of the chest revealed the absence of broncho-pneumonia, and by method of exclusion the diagnosis of diaphragmatic pleurisy was arrived at. The after history of the case fully confirms the diagnosis.

The movability of the pleuritic exudate is the subject of a study by Baccelli. He thinks the best method by which to demonstrate the movement of the exudate is to examine the patient first sitting, then prone. The sero-fibrinous exudation changes its position with that of the body when it is small in quantity or when medium in amount, and no strong pressure on the parenchyma of the lungs is occasioned. The thinserous fluids change their position faster than the sero-fibrinous.

In the diagnosis of consolidation of the lung from serous or purulent effusions, Forchheimmer advises the serious consideration of the age of the patient. He seldom finds serous effusion under four years of age, empyema being most common at that period of life. He has been unable to utilize some of the fine auscultatory signs that have been put down, for instance the greater transmission of sound in cellular fluids ; they seem to him without value in practice, especially in children.

The treatment of pleurisy in children is discussed by Plicque. In the early treatment calomel and digitalis are recommended, and a protest against the abolition of the poultice is recorded, which he believes aids absorption and prevents the progress of the disease to the purulent stage. Tapping should always be done at first with the aspirator, an antiseptic fluid being used to wash out the cavity. Fluid should always be withdrawn slowly and gently and the introduction of the antiseptic solution—lukewarm boracic lotion is the best for the purpose—performed with equal care and deliberation. No fear need be felt in using large quantities of fluid to flush out the pleura ; as much as five or six litres have been employed. If tapping fails to prevent the reformation of purulent fluid, an incision must be made and irrigation carried out in the same way. The anterior axillary line should be selected for the incision instead of the posterior, as in the adult. In case of encysted fluid the incision must be directly over the point of localization. Frequent and cautious washing out of the cavity is advised in after-treatment. Estlander's operation is rarely necessary in the case of infants.

The question of the treatment of purulent effusions is still much discussed, especially in Germany, where physicians are generally opposed to the operation for empyema, while surgeons favor it. Bozzolo affirms that in his experience, simple puncture for empyema has always failed to cure ; here costal resection is always indispensable.

Massage for pleuritic effusions has an advocate in Poliateon, who has employed massage of the chest in ten cases of primary pleuritic exudations, seven being purely serous and three sero-fibrinous. The manipulations were made in the direction of lymphatics of the affected region, radiating towards the axilla. The treatment was commenced by light effleurage, but soon brisk rubbing was resorted to, followed by clajottement. The sittings, which took place daily, lasted from ten to twenty minutes. Under this method the exudation disappeared in from eight to twenty days, while the other symptoms disappeared in from nine to thirty-five days. Massage of the thorax, in cases of pleuritic exudation, acts as a counter-irritant similarly to blisters and the cautery, but has the advantage that it can be practiced every day.

Aside from its irritant effects, massage relieves the chest pains, invigorates the muscles, and augments the volume of the respiratory movements ; an action which exerts marked influence on the absorption of the exudation and the distention of the compressed lung.

### THE SALYCILATE OF SODA IN SEROUS PLEURISY.

Germain See pointed out that the salycilate of soda, while curing the primary disease in acute articular rheumatism, prevented serous inflammation. Aufrecht showed that this is an excellent remedy for idiopathic serous pleurisies, whatever their origin. This was subsequently confirmed by Eichhorst, Maragliano, Stiller, Dozewiecki, Letz, Deri and Talamon. According to Letz and Talamon the curative effect of salycilate of soda in serous pleurisy is not less specific than in rheumatism. Stritzover reports eight cases of exudative pleurisy in which he gave the drug internally in one gramme doses three times a day. As a precaution against collapse, the dose was always given after meals. Each dose being followed by a draught of good wine. In seven cases the temperature rapidly fell to normal, and the subjective condition improved, while in about a week dyspnœa was relieved, and the area of dullness lessened. Every one of the patients completely recovering in about eighteen days. In the eighth case the treatment of four days' duration utterly failed. The symptoms pointed to empyema. An exploratory aspiration was made, and a sero-purulent fluid escaped. The patient was ultimately cured by excision of a rib. The doctor is of the opinion that this is a most valuable remedy in serous pleurisy, which at the same time affords a reliable means of determining the character of pleural effusion, that is, of the differentiating serous from sero-purulent or purulent pleurisy.

Antipyrine in pleuritic effusions was the subject of a communication by Clement. His attention had been called to this subject three years ago, and his observations were based upon some twenty cases. He notes the upper limit of dullness with lunar caustic, and can thus note the slightest variation in the amount of the fluid. Whether fever be present or not he at once administers antipyrine without any other medication whatever. In all cases there was on the following day, or at least the day after, a marked reduction in the height to which the dullness reached. In some cases the dullness disappeared in forty-eight hours ; in two or three the fluid was completely absorbed in twenty-four hours. In no case was the effect delayed beyond four days. The dose was one gramme every four hours. He thinks antipyrine has a specific action on nearly all inflammatory processes, and it is to this that he attributes its beneficial effect in pleural effusion. This remedy failed when the effusion was purulent or bloody.

